# Acute intestinal obstruction secondary to ingested foreign body in an adult with autism spectrum disorder: A rare case report and review of literature

**DOI:** 10.1002/ccr3.8759

**Published:** 2024-04-11

**Authors:** Suraj KC, Rakesh Kumar Gupta, Abhijeet Kumar, Bhawani Khanal, Samiksha Lamichhane, Amrit Buhusal, Vijay Pratap Sah, Sanjok Bartaula, Injmamul Haque Raki, Raghav Jindal

**Affiliations:** ^1^ Department of General Surgery BPKIHS Dharan Nepal; ^2^ Department of Radiodiagnosis and Imaging BPKIHS Dharan Nepal

**Keywords:** autism, foreign body, intellectual, laparotomy, obstruction

## Abstract

**Key Clinical Message:**

Foreign body ingestion is common in pediatric age group however can be found in any age group with intellectual disability and neurodevelopmental delay. There is usually a delay in presentation and interventions following foreign body ingestion in patients with neurodevelopmental delay, leading to increased morbidity, mortality, and complications owing to inability of such patients giving relevant history. Most ingested foreign bodies naturally pass through the digestive tract without untoward effects. Only a few patients may require surgical interventions. Principle of management should be to reduce anxiety among patients and their visitors. Speedy recovery is enhanced so that they can return to their familiar environment soon.

**Abstract:**

Foreign body ingestion is common in pediatric populations and may be found in any age group with intellectual disability and neurodevelopmental delay. As the patient cannot give a clear and relevant history, there is usually a delay in presentation and interventions following foreign body ingestion in patients with neurodevelopmental delay, leading to increased morbidity, mortality, and complications. Most foreign bodies pass through the digestive system without any complications, and very few require surgical intervention. The goal should be to reduce anxiety among patients and their visitors and to enhance speedy recovery so that they can return to their familiar environment soon. Here we report a case of Acute intestinal obstruction secondary to ingestion of the head portion of a doll which was managed with emergency laparotomy with enterotomy and removal of foreign body in a 16 years female with Autism Spectrum Disorder.

## 1 INTRODUCTION

Pediatric emergencies usually receive a lot of cases of foreign body ingestion, as this event is more likely to occur in pediatric age group populations. The American Association of Poison Control Centers in 2002 reported that 75% of the >116,000 FB ingestions reported occurred in children aged ≤5 years.^1^ Sometimes patients with neurodevelopmental delay may present with features of acute intestinal obstruction, as in our case. Foreign body ingestion should always be in our differentials, as they are usually silent and cannot give a relevant history. Most ingested foreign bodies naturally pass through the digestive tract without untoward effects.^2^ Only a few patients (10%–20%) may require surgical interventions.^3^ Patient cannot give a clear and relevant history, there is usually a delay in presentation and interventions following foreign body ingestion in patients with neurodevelopmental delay which was consistent with our case where a 16‐year‐old female diagnosed with autism spectrum disorder (ASD) presents with features of intestinal obstruction secondary to ingestion of the head portion of a doll which was managed with emergency laparotomy with enterotomy and removal of foreign body under general anesthesia. This sort of presentation is rare at our center, so it is being reported.

## 2 CASE HISTORY

A 16‐year‐old female presented to the emergency department with a history of being unable to pass stool and flatus for 4 days. She is a known case of autism spectrum disorder, so her history was given by her parents. They also complained of multiple episodes of bilious vomiting for 1 day. For the past 4 days, the child has been screaming episodically due to abdominal pain. They gave no history of fever, vomiting, abdominal distension, shortness of breath, or cough. She was diagnosed with autism spectrum disorder and was on medication. She was on her regular follow‐up with the psychiatric department. There was no history of other comorbidities and prior hospitalization.

### 2.1 General physical examination

On physical examination, she was conscious and well‐oriented to time, place, and person. She was hemodynamically stable.

### 2.2 Systemic examination

The abdomen was s non‐distended with tenderness over the umbilical region. Bowel sounds were present. A digital rectal examination revealed a collapsed rectum with stool staining the fingers.

## 3 METHODS

### 3.1 Differential diagnosis

With above findings we have kept constipation, intussusception, and foreign body ingestion as our differentials.

### 3.2 Investigations

**FIGURE 1:**
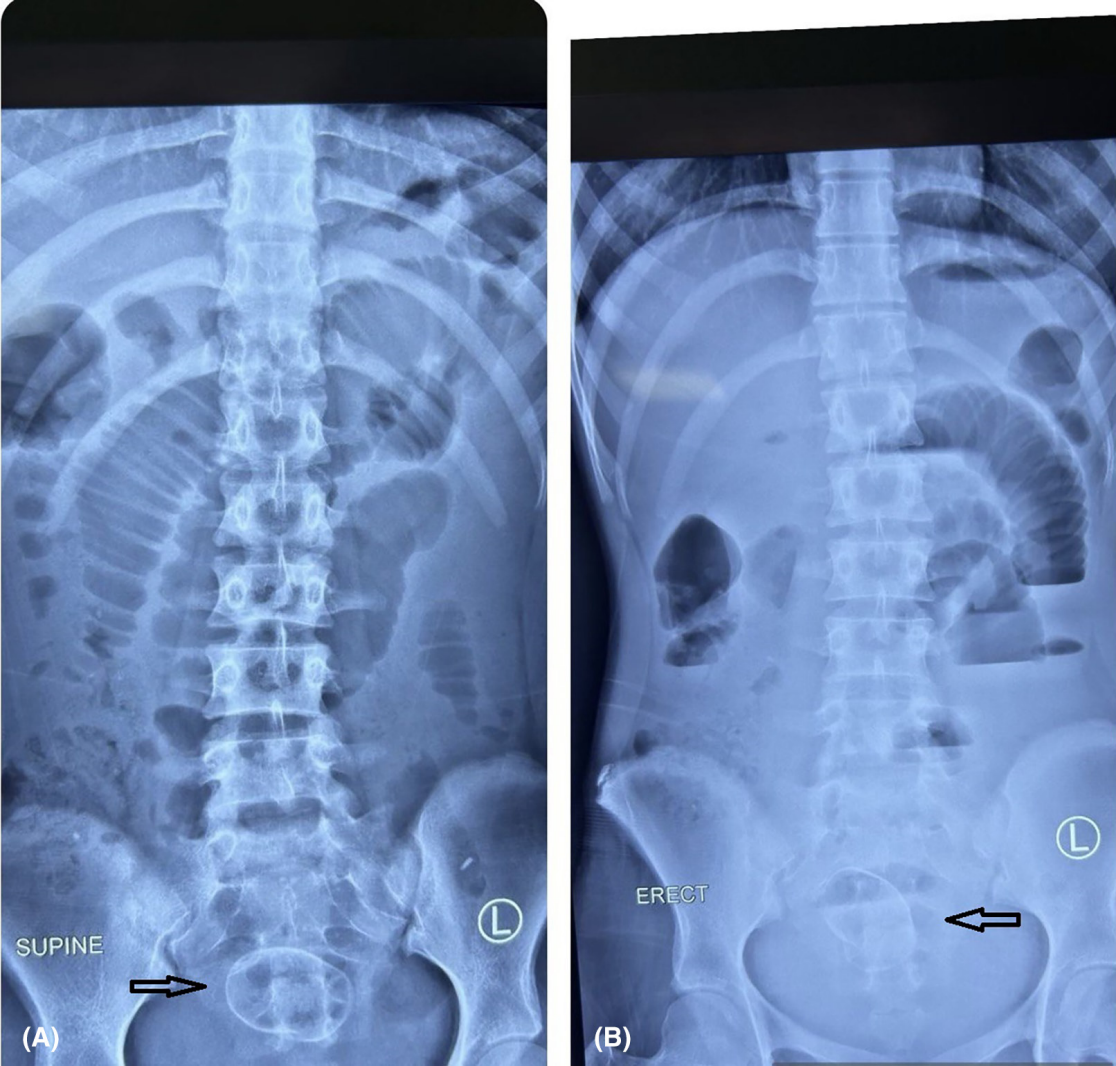
(A) x‐ray of abdomen and pelvis in supine view with dilated bowel loops likely jejunum. There is well defined oval shaped peripherally radio opaque with central radioluscency noted at the pelvis in erect view likely foreign body (black arrow). (B) x‐ray of abdomen and pelvis in erect view showing dilated bowel loops and multiple air fluid level. There is well defined oval shaped peripherally radio opaque with central radioluscency noted at the pelvis in erect view likely foreign body (black arrow).

**FIGURE 2:**
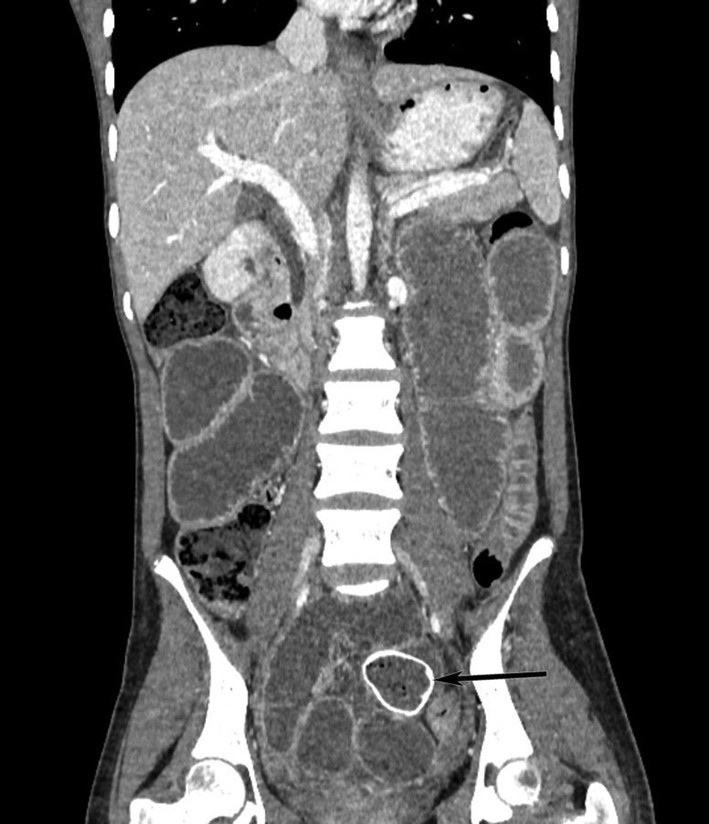
Coronal Section of CECT shows well defined oval peripherally hyperdense structure of size 3.8 × 2.7 cm is noted in proximal ileum causing dilatation of proximal bowel loops.

**FIGURE 3:**
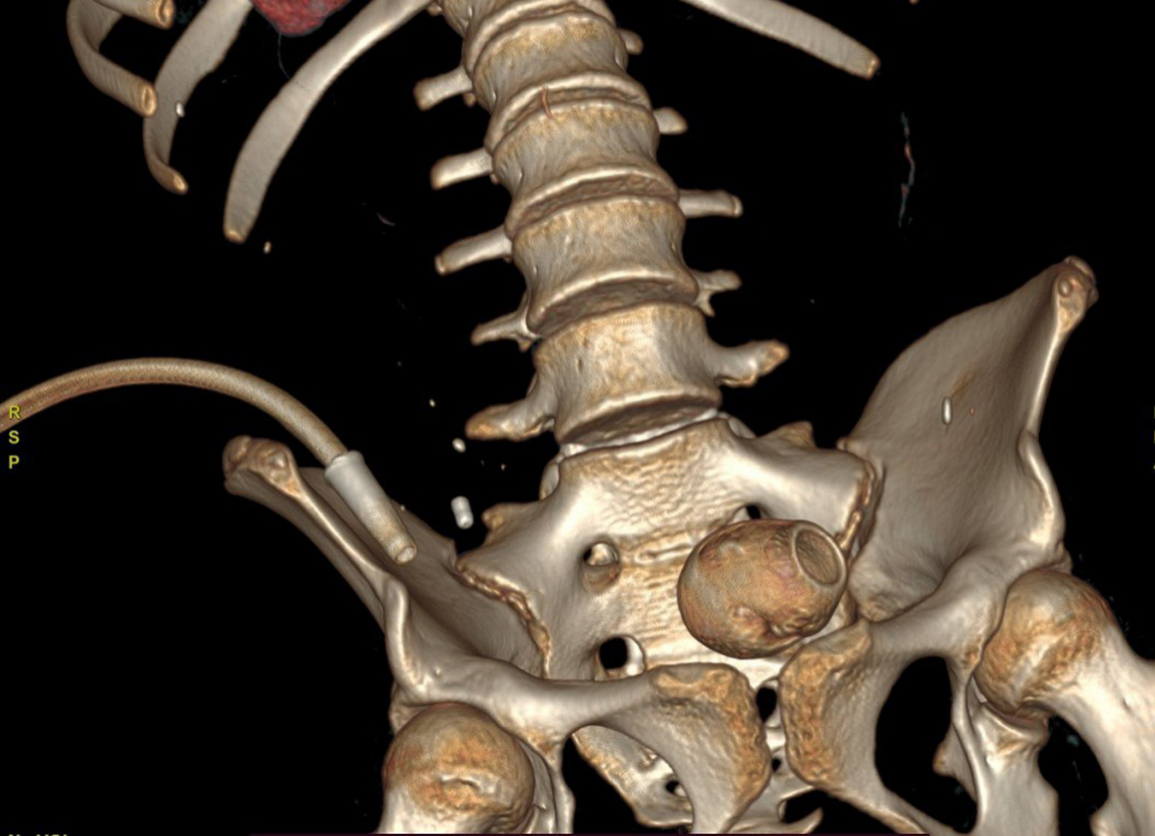
3D volume rendering image showing foreign body in pelvis.

Complete blood counts, renal function test. liver function test were with in normal limits. C reactive protein was also with in normal range x‐rays of the abdomen and pelvis on an erect and supine view showed a dilated small bowel loop with multiple air fluid levels suggestive of intestinal obstruction. There was well defined oval shaped peripherally radio opaque with central radioluscency noted at the pelvis in erect view likely foreign body (Figure 1). Contrast‐enhanced computed tomography shows an oval‐shaped well‐defined soft tissue density structure with a hyperdense rim measuring about 4.4 × 3.4.3.3 cm (AP X TR X CC) in the proximal ileum located at the pelvis, a likely transition point with multiple dilated proximal jejunal loops, and an air fluid level within a likely foreign body in the proximal bowel with small bowel obstruction (Figures 2 and 3).

### 3.3 Treatment

**FIGURE 4:**
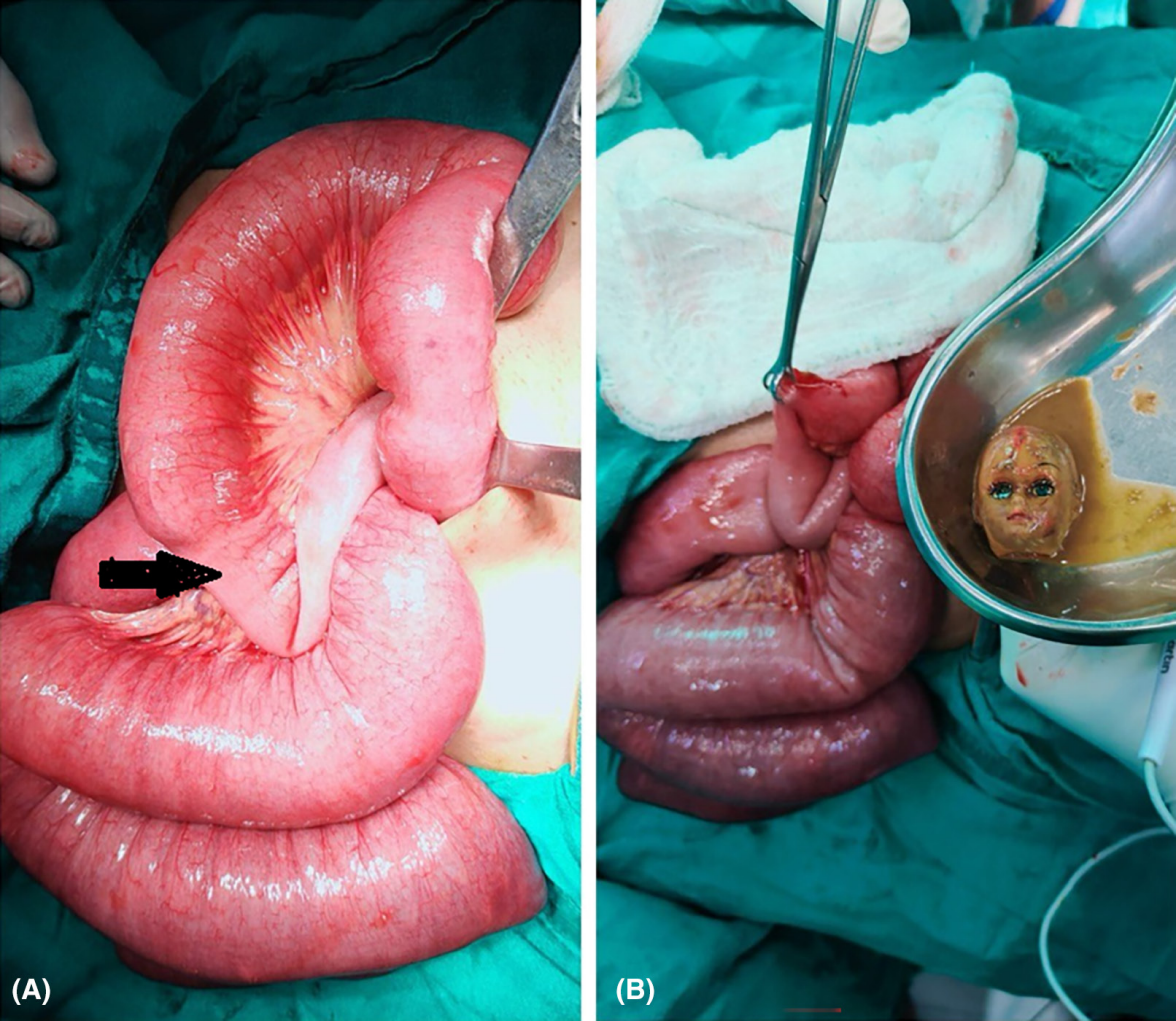
(A) dilated bowel loop with transition point (arrow). (B) Image showing the foreign body and enterotomy.

She was diagnosed as a case of Acute intestinal obstructions secondary to foreign body ingestion so she underwent exploratory laparotomy with enterotomy and removal of a foreign body under general anesthesia (Figure 4). Intraoperative findings were a grossly dilated jejunal loop with a transition point at the proximal ileum. The foreign body that is, doll's head, was located 100 cm proximal to the Ileocecal valve. Primary repair of the enterotomy was done with PDS 3–0. An abdominal drain was placed.

### 3.4 Outcomes and follow up

The postoperative period was uneventful. Oral feeding was started on the second postoperative day, which she tolerated well. The drain was removed on the third postoperative day. She was discharged on the third postoperative day on oral medication. She followed up on 10th postoperative period for stiches removal. The wound was healing and she was recovering well.

## 4 DISCUSSION

Autism spectrum disorder (ASD) is a neurodevelopmental disorder characterized by deficits in social communication and the presence of restricted interests and repetitive behaviors.^4^ A team of specialists, including psychologists, occupational therapists, and speech‐language pathologists, is required for the assessment and diagnosis of autism spectrum disorder.^5^ In our case, the child lacked verbal communication and exhibited restricted interests and repetitive behaviors. The pediatric population is more vulnerable to foreign body ingestion, with a peak incidence between the ages of 6 months and 6 years.^6^ Psychiatric disorders and intellectual disability/neurodevelopmental delay could be the reasons for ingestion in the older population.^4^, ^7^ In our case, the patient does not fall into the pediatric population. The most commonly ingested object is coins in more than 60% of children, followed by toys at 10% and jewelry at 7%.^2^ 80%–90% of ingested foreign bodies naturally pass through the digestive tract within 4–6 days without untoward effects, while endoscopic removal is indicated in 10%–20% of cases.^3^ The esophagus, pre‐pyloric region, the ligament of Treitz, the ileocecal valve, and the rectosigmoid junction are the narrowest structures in the digestive tract where obstruction is likely.^8^ Foreign body intestinal perforation accounts for only 1% of all intestinal perforations, while the rest of the foreign body passes without any complications.^9^ The ingestion of batteries and sharp objects such as bones and needles is highly associated with perforations.^10^ Perforation is most common in the sigmoid colon, followed by the rectum and distal ileum, as per earlier studies.^9^, ^11^ Higher rates of surgery, perforation, and mortality in the group of patients with intellectual disability‐neurodevelopmental delay as compared to the normal population are due to delays in presentation and interventions.^12^ Radio‐opaque foreign bodies are commonly diagnosed by x‐rays, while diagnostic images, such as contrast x‐rays and CT, may be useful in radio‐opaque and in children as well.^13^ Hospital admission is an overwhelming sensory and cognitive experience for autistic children,^14^ so efforts must be made to reduce patient and family anxiety and ensure a speedy discharge to their familiar environment.^15^ Surgical management is indicated in very few patients. Multiple failed endoscopic attempts and complications that cannot be treated endoscopically are relative indications for surgical intervention.^16^ In our case the foreign body was located at was located 100 cm proximal to the Ileocecal valve. due to limited resources and unavailability of endoscopic facilities we have opted surgical intervention without any delay.

Our case is unique in the sense that she has ingested a doll's head (7%), which is rare according to the literature review. Additionally, there has been a need for surgical intervention (80%–90%) of foreign bodies pass through the digestive tract naturally in our case, which is quite uncommon according to the literature review.

## 5 CONCLUSION

Foreign body ingestion is more likely to occur due to neurodevelopmental delay in older age. Most foreign bodies pass easily without any complications. There is usually a delay in presentation and interventions in the case of ingestion by a patient with a neurodevelopmental disorder. Measures should be taken to reduce anxiety among patients and their families to ensure a speedy recovery and return to a familiar environment as soon as possible. A suspicion of foreign body ingestion should always be kept in mind when dealing with pain abdomen and bowel obstruction in patients with neurodevelopmental delay, including Autism spectrum disorder as they have poor verbal communication and could provide us the accurate and reliable history as seen in our case.

## AUTHOR CONTRIBUTIONS

**Suraj KC:** Conceptualization; data curation; formal analysis; investigation; supervision. **Rakesh Kumar Gupta:** Conceptualization; data curation; methodology; software; supervision; writing – review and editing. **Bhawani Khanal:** Conceptualization; methodology; supervision; visualization. **Abhijeet Kumar:** Conceptualization; formal analysis; methodology; resources; supervision. **Samiksha Lamichhane:** Conceptualization; investigation; software; supervision. **Amrit Bhusal:** Formal analysis; methodology; supervision; visualization. **Vijay Pratap Sah:** Data curation; methodology; resources; validation. **Sanjok Bartaula:** Investigation; project administration; software; visualization. **Injmamul Haque Raki:** Data curation; formal analysis; resources; software. **Raghav Jindal:** Conceptualization; formal analysis; resources; software.

## FUNDING INFORMATION

None.

## CONFLICT OF INTEREST STATEMENT

None.

## ETHICS STATEMENT

This study has been performed according to the declaration of Helsinki.

## CONSENT

Written informed consent was obtained from the patient party to publish this report in accordance with the journal's patient consent policy.

## Data Availability

The data that support the findings of this study are available from the corresponding author upon reasonable request.
